# Supplementary material for: The interactome of intact mitochondria by cross-linking mass spectrometry provides evidence for coexisting respiratory supercomplexes
Source: Mol Cell Proteomics. 2017 Dec 8;17(2):216–32. doi: 10.1074/mcp.RA117.000470 (PMC5795388; doi:10.1074/mcp.RA117.000470)
Supplement: Supplemental Data [file supp_RA117.000470_133922_0_supp_23978_fzffwf.zip › spectra_annotation/mito_DR_spectra_annotation/229-1-10-1-20-1.pdf]

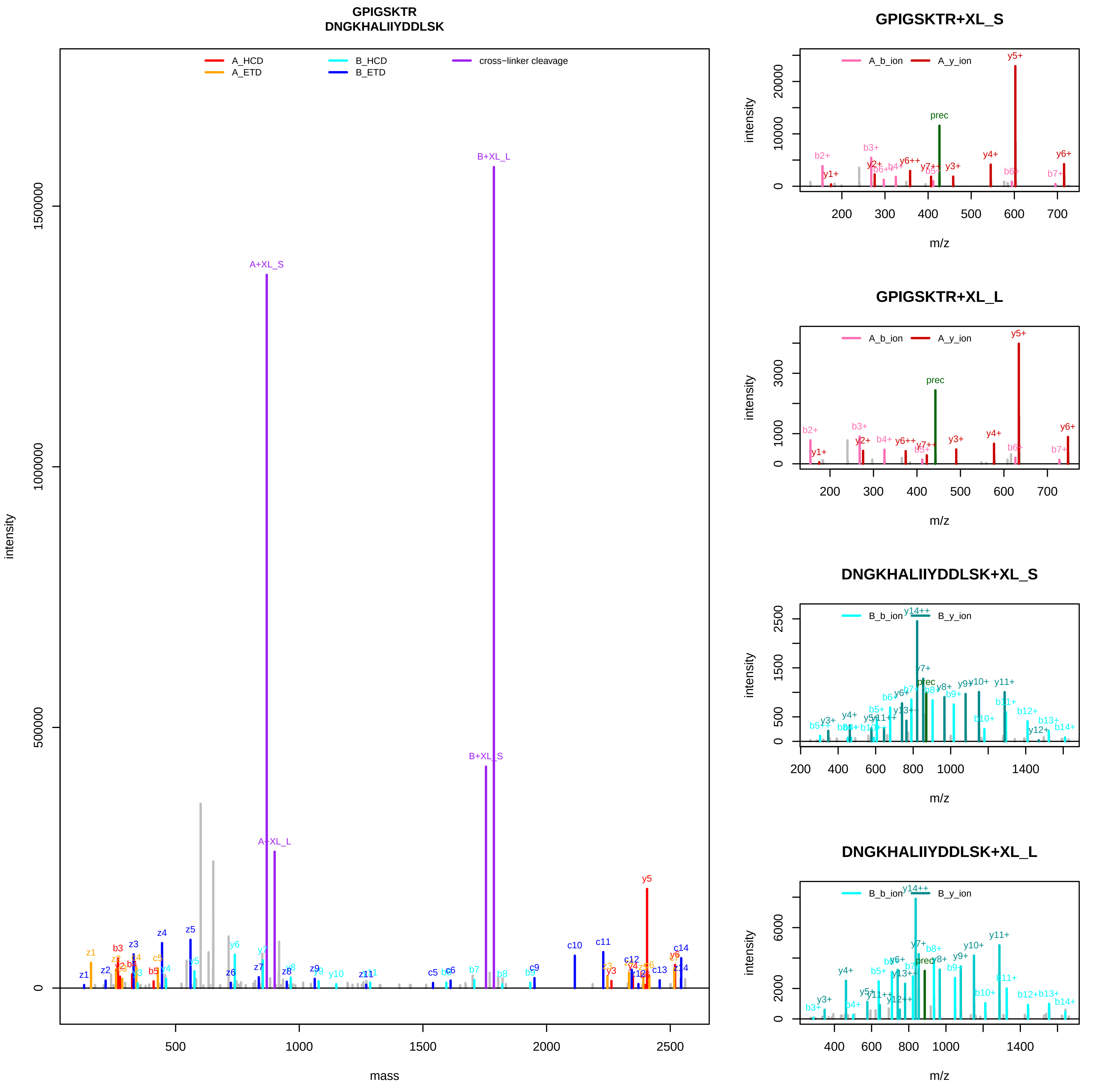

GPIGSKTR
DNGKHALIIYDDLSK
A_HCD
A_ETD
B_HCD
B_ETD
cross−linker cleavage
intensity
mass
0
500000
1000000
1500000
500
1000
1500
2000
2500
A+XL_S
A+XL_L
B+XL_L
B+XL_S
y5
c10
c11
c12
c13
c14
z1
z2
z3
z4
z5
z6
z7
z8
z9
z11
z14
b3
b5
c5
c6
c9
y6
y7
y8
y10
b7
b8
b9
GPIGSKTR+XL_S
GPIGSKTR+XL_L
DNGKHALIIYDDLSK+XL_S
DNGKHALIIYDDLSK+XL_L
A_b_ion
A_y_ion
B_b_ion
B_y_ion
prec
m/z
0
10000
20000
1000
3000
500
1500
2500
2000
6000
200
300
400
500
600
700
800
1000
1400
y5+
y14++
y11+
